# Impact of the Dinner Tonight Healthy Cooking School on Participants’ Nutrition Knowledge and Cooking Confidence

**DOI:** 10.3390/nu17101668

**Published:** 2025-05-14

**Authors:** Sumathi Venkatesh, Odessa E. Keenan, Morium B. Bably

**Affiliations:** 1 Department of Nutrition, Texas A&M AgriLife Extension Service, College Station, TX 77840, USA; 2 Texas A&M AgriLife Research, El Paso, TX 79927, USA; morium.bably@ag.tamu.edu

**Keywords:** overweight and obesity, healthy cooking, nutrition education

## Abstract

**Background/Objectives**: Nearly three-quarters of American adults are overweight or obese, leading to serious health challenges. Poor nutrition is a major contributor, highlighting the need for effective nutrition education and cooking interventions. This study evaluated the impact of the Dinner Tonight Healthy Cooking School (DTHCS) on improving nutrition knowledge and cooking confidence among participants. **Methods**: A secondary data analysis was conducted on self-reported retrospective surveys collected from 7932 individuals across 64 Texas counties between September 2017 and March 2024. The surveys assessed changes in participants’ understanding of key nutrition concepts (rated using a four-point scale from poor to excellent) and their confidence in cooking healthy meals (rated using a four-point scale from none to high) before and after attending the program. **Results**: The findings showed significant improvements in both nutrition knowledge (from fair to good *p* < 0.001) and cooking confidence (from low to moderate *p* < 0.001) following program participation. **Conclusions**: These results highlight the vital role of nutrition and culinary education in addressing the growing obesity epidemic and reducing the risk of diet-related chronic conditions. Given the positive program outcomes, DTHCS has the potential to inform future studies and guide successful planning and implementation of community-based cooking programs for adults to promote healthier eating behaviors.

## 1. Introduction

Obesity prevalence has surged in the United States over the past three decades, with about 172 million adults aged 25 and older classified as overweight or obese [1]. If current trends continue without preventive measures, an additional 41.4 million adults could be affected by 2050 [1]. Obesity increases the risk of type 2 diabetes [2], cardiovascular disease [3], hypertension [4], and other serious health complications. Additional challenges include disability [5], psychological distress, reduced quality of life [6,7,8], and significant financial burdens [9].

Poor dietary behaviors such as low fruit and vegetable intake, large portion sizes, frequent snacking, and high intake of energy-dense foods, added sugars, and fast-food consumption has been shown to be strongly associated with weight gain and obesity [10,11,12,13,14,15]. Frequent consumption of foods away from home has become common due to social and economic factors including busy lifestyle, long work hours, and increased availability of dining and delivery options, and is associated with lower diet quality and higher caloric intake compared to home cooked meals [16,17].

Previous studies have identified several personal barriers, including time constraints, limited cooking skills or confidence, lack of motivation and interest in cooking, insufficient knowledge of healthy ingredients and recipes, budgetary concerns, limited access to healthy ingredients, and lack of appropriate kitchen tools and equipment [18]. Such barriers may have a considerable impact on the dietary behaviors of adults [19]. Moreover, limited nutrition knowledge is a key contributor to overweight and obesity, but this can be addressed through effective nutrition education programs that promote healthy dietary behaviors [20,21,22].

Food and nutrition literacy plays a key role in improving dietary decision-making and overcoming barriers to healthy eating [23]. Community-based practical cooking education programs can help overcome these barriers [24] by equipping individuals with essential skills and knowledge, thereby promoting healthier food choices and enhancing overall diet quality [25,26]. These programs should be designed to effectively educate and encourage adults to improve their food behaviors and cooking practices, ultimately supporting weight management [19]. Specifically, such programs should feature recipes with fewer ingredients that are healthy and affordable, minimal equipment, and shorter cooking times; include take home resources; and incorporate nutrition talking points [27]. While the existing literature has documented the importance of food literacy in promoting healthy food consumption, the effectiveness of cooking interventions in addressing barriers such as lack of nutrition knowledge or cooking confidence has not been extensively evaluated. Thus, this study aims to examine the effectiveness of Dinner Tonight Healthy Cooking Schools (DTHCS), a community-based cooking education program on participant’s understating of healthy foods and confidence in making healthy food choices.

### Dinner Tonight Healthy Cooking School

The Texas A&M AgriLife Extension Service developed DTHCS in 2009 with a team of Extension professionals to educate busy families about healthy cooking and provide nutritious, affordable recipes. DTHCS are interactive cooking events that emphasize nutrition, food safety, meal planning, and cooking techniques in a casual, engaging atmosphere. There are no specific eligibility requirements for participation. DTHCS events last approximately two hours and include live recipe demonstrations. The foundational curriculum includes implementation guidelines, recipe booklets, educational content, marketing materials, participant resources, and an evaluation survey. Client-facing materials, such as recipe booklets, are translated and available in both English and Spanish. Any additional implementation considerations, educational content, and marketing materials are made available as they are developed.

A new edition of the recipe booklet or a supplemental curriculum is developed approximately every 18 months, each focused on a specific theme such as diabetes management, Mediterranean cooking, seafood, wild game, batch cooking, cooking with pantry staples, and emergency preparedness. All recipes align with the Dietary Guidelines for Americans by limiting saturated fats, sodium, and added sugars, and meeting minimum recommendations for beneficial nutrients such as fiber, Vitamin D, Calcium, Iron and Potassium [28]. Recipes were designed to be simple, healthy, and affordable, using minimal ingredients and equipment, and requiring shorter cooking times. Each recipe has been tested, refined, and standardized for consistency and ease of preparation. DTHCS features a vast collection of recipes across several cookbook editions and on the program website (https://dinnertonight.tamu.edu/). The website also offers additional resources, including video demonstrations and nutrition facts.

## 2. Materials and Methods

### 2.1. Data Source

Secondary data were requested from the Office of Data and Accountability (ODA) at Texas A&M AgriLife Extension Service in November 2024 for further analysis to investigate the study objective. The Texas A&M University Institutional Review Board approved the study to analyze and publish secondary data collected from DTHCS between September 2017 and March 2024.

### 2.2. Educator Training

DTHCS was primarily delivered by Extension educators, who are trained professionals across the state that provide educational outreach and resources to local communities. They served as the primary instructors, leading recipe demonstrations and delivering educational content. Each year, Extension educators receive training from a program manager to ensure that participants across the state receive a consistent, high-quality educational experience, regardless of the region in which they attend an event. On average, two training sessions are offered annually and may be delivered virtually or in person, depending on the availability of resources and identified needs. The first training covers the general structure of the DTHCS and specific editions, emphasizing program fidelity through adherence to implementation guidelines, proper utilization of program materials, marketing strategies, and evaluation procedures. The second training focuses on the logistics of implementing a successful food demonstration, including planning, organizing, and execution. Additional training opportunities are offered based on request from program and state leaders who are responsible for Extension programming and administration.

### 2.3. Program Implementation

To strengthen community partnerships, enhance program visibility, and offer culinary expertise, Extension educators are encouraged to involve local guest chefs and adapt DTHCS to meet the unique mees of their communities. Extension educators determine program sites, recruitment strategies, and participation costs based on their knowledge of operating within their communities. The cost of implementing a DTHCS event can vary widely depending on factors such as venue, marketing strategy, food sampling approach, and the availability of local resources. Extension educators collaborate with local organizations and businesses to gain support, secure resources, and assist with participant recruitment. Venue-related considerations include the anticipated number of participants, types of recipes to be demonstrated, food distribution method, involvement of vendors or community partners, and overall budget constraints. A minimum registration fee of USD 10 is recommended to cover the cost of demonstrations, food tastings, and the take-home recipe booklet. The recipe booklets also featured key information on nutrition, food safety, meal planning and cooking techniques discussed during the live demonstrations. Participants were encouraged to sign up for weekly digital newsletters and following social media accounts to continue to receive reinforcing educational messaging and relevant recipes.

### 2.4. Program Evaluation

Program participants completed a retrospective post-survey at the end of the event. The survey collected demographic information and self-reported health status, rated on a five-point scale from excellent to poor. For analysis, responses of excellent, very good, and good were combined into one category, while fair and poor were combined into a second category. Participants also reported whether they engaged in moderate or vigorous physical activity in the past month (yes/no), the number of meals consumed outside the home, and their weekly spending on these meals. They were asked to indicate what portion of their dinner plate was filled with fruits and vegetables. Responses were grouped into two categories, with one indicating more than ½ and the other less than ½.

The survey also measured the frequency of fruit, vegetables, soda, and sugar sweetened beverage intake using a seven-point scale from never to four or more times a day. For analysis, fruit and vegetable intake responses were grouped as ≤3 times a day and >3 times a day. Responses for soda and sugar sweetened beverage intake were grouped as ≤3 times a week and >3 times a week. Participants rated their understanding of the benefits of meal planning, the amount of sodium, fat, calories in the foods they prepare, and the impact of food on health before and after the event, using a four-point scale from poor to excellent. Similarly, they rated their confidence level in meal planning and healthy food preparation using a four-point scale from none to high. For analysis, these items were treated as continuous variables.

Evaluations were administered using scannable paper-based surveys, which were mailed by the Extension educators to ODA, where they were managed and stored. To conduct the data analysis for this study, secondary data were obtained from ODA to investigate the study objectives. Participants’ demographic characteristics and current dietary and physical activity behaviors were analyzed using descriptive statistics. Paired *t* tests were conducted to examine any improvements in participants’ nutrition knowledge and their confidence in healthy food preparation before and after the event.

## 3. Results

### 3.1. Participant Characteristics

Participant characteristics are reported in Table 1. From September 2017 to March 2024, DTHCS reached 7932 participants statewide representing 64 counties in the Central, East, North, South, Southeast, and West regions of Texas. Most program participants were from East, Southeast, and Southern Texas (88.2%). The age range of participants was 10 to 94 years and predominantly consisted of middle age and older adults. Program participants were mostly female (73.2%, n = 5804) and were mostly Hispanic or white (constituting more than 80% of the total sample).

### 3.2. General Health, Physical Activity, and Nutrition

Participants’ self-reported health status, physical activity, and food behaviors are summarized in Table 2. Two-thirds of participants (n = 6104) rated their general health as excellent, very good, or good. About 60% engaged in moderate or vigorous physical activity within the past week. Approximately 75% of program participants reported never/rarely or occasionally consuming meals outside the home and typically spending under USD 150 per week on such foods. Over half of program participants (n = 4512) indicated that fruits and vegetables comprised less than half of their dinner plate. Additionally, fewer than one-fifth of participants consumed fruit (12.9%) and vegetables (18.9%) at least three or more times a day. The majority of participants reported moderate consumption of regular soda or sugar sweetened beverages (such as fruit punch, fruit drinks, sweet tea, or sports drinks), with occasional intake averaging 0–3 times per week, rather than daily.

### 3.3. Nutrition Knowledge and Confidence in Healthy Cooking

Table 3 shows changes in participant’s nutrition knowledge and confidence in healthy food preparation before and after attending DTHCS. Participants reported significant improvements (*p* < 0.001) in their understanding of the benefits of meal planning, as well as their knowledge of sodium, fat, and calorie content of foods, and the impact of diet on health, after attending DTHCS. Similarly, participants self-reported significant improvements (*p* < 0.001) in their confidence in meal planning, modifying recipes to reduce sodium, fat, and calories, selecting produce from demonstrations, and safely storing and preparing fruits, vegetables, and meats from the demonstrations after attending the program.

## 4. Discussion

This study aimed to assess the effectiveness of DTHCS, a community-based cooking program, in enhancing participants’ understanding of healthy foods and boosting their confidence in making healthier food choices. The program reached 7932 participants in 64 counties and documented significant improvements in both nutrition knowledge and cooking confidence among participants. Most of our program participants were women, which is typical for a Cooperative Extension community cooking program [24,26].

Our sample consisted of individuals who reported good to excellent general health and demonstrated healthy food and exercise behaviors at baseline, such as regularly engaging in moderate to vigorous physical activity, avoiding outside meals, and consuming soda and sugar sweetened beverages in moderation. Studies have shown that individuals who report good to excellent health are likely to engage in health-promoting behaviors due to their health awareness and motivation to adopt healthy lifestyle habits [27,29,30]. The Health Belief Model, a psychological framework used to understand health behaviors, suggests that individuals are more likely to engage in health-promoting behaviors if they perceive themselves as susceptible to health issues and recognize the benefits of preventive actions [31]. Furthermore, studies have shown that healthier lifestyle habits are linked to higher well-being, including effective weight management [32,33].

Given that our sample predominantly consisted of mid-age to older adults, it is possible that they are also more inclined to adopt healthy behaviors due to medical advice or lifestyle changes aimed at managing existing chronic conditions [34]. Fruit and vegetable intake, however, was low in our sample, with more than 80% of our participants consuming them less than three times a day, which is consistent with the findings of Wang et al. [35]. Furthermore, when asked about their dinner intake, only 40% of our study participants reported meeting the recommendation to make half their meal plate fruits and vegetables. The Dietary Guidelines for Americans and the MyPlate visual guide recommend this for every meal [36]. These findings suggest that although participants had interest in and intention to adopt healthy behaviors, they may have been unsure of incorporating fruits, vegetables, and other healthy ingredients into their cooking, possibly due to limited nutrition knowledge or a lack of confidence in preparing healthy meals [19,37]. Given this, cooking interventions such as the DTHCS, which offer a variety of healthy recipes and emphasize the inclusion of plenty of fruits and vegetables, could play a key role in improving healthy food intake [38].

In our study, participants demonstrated an increase in nutrition knowledge, improving from fair to good after attending the program. These results are consistent with the findings from other studies, which also reported increased nutrition knowledge and supported the effectiveness of nutrition education programs across various populations [25,26,39]. Additionally, our study documented an improvement in participants’ confidence in healthy cooking, with confidence rising from low to moderate after attending the program. These results align with previous research, which has similarly reported increased self-efficacy, motivation and confidence among participants following nutrition and cooking intervention [1,19].

Sustainability is key to the long-term success of health programs like DTHCS. To promote sustainability of the program within Texas, the DTHCS program curriculum and its subsequent editions are available to all Extension educators as part of their county programming materials. Training on the program curriculum is provided at no cost and is included in internal professional development opportunities. The DTHCS curriculum is regularly updated to maintain its relevance to communities and ensure new and returning clients are satisfied. The DTHCS is an adaptable program built for sustainability, with a focus on partnering locally to engage community stakeholders in its success. The Dinner Tonight program also builds and maintains state level partnerships that foster sustainability for the program at large. Additionally, encouraging program fees for participants helps sustain the program at both the local and state level.

Our study had several strengths and limitations. The large dataset strengthens the application of the findings and ensures representation from all regions of Texas. Also, DTHCS is a one-day event, requiring no long-term commitment, making it accessible for busy participants. The program featured live cooking demonstrations with tastings and offered a wide range of recipes across multiple editions of the DTHCS cookbooks and on the website. All recipes were designed to be nutritionally balanced, affordable, and easy to prepare.

Our sample predominantly consisted of women and mid-to-older adults, which may limit the generalizability of the findings to the food behaviors and nutrition knowledge of younger adults. The study relied on self-reported dietary and health data, which may be subject to over- or underestimation of actual values due to response bias. We did not collect dietary data using a formal dietary assessment tool, such as a food frequency questionnaire or a 24 h dietary recall. This approach reflects the nature of Extension programming and evaluation, which is primarily focused on program assessment and internal reporting. Implementing more robust evaluation methods would have provided a deeper understanding of the nutrition and food behaviors of this target population.

Furthermore, our survey requested only the daily frequency of fruit and vegetable consumption and did not account for the quantity consumed at each instance. A key limitation of using a retrospective survey is the potential for recall bias. Future studies may consider conducting a three-month follow-up assessment to capture more accurate and reliable results, thereby strengthening the evidence for dietary changes resulting from program participation. Lastly, since our analysis relied on secondary data, we were limited in our ability to control for external factors, such as pandemic-related challenges. The program period overlapped with the COVID-19 pandemic, which led to a system-wide shift to virtual and remote programming. Extension educators followed U.S. federal, state, and county regulations regarding in-person gatherings, including educational events. Best practice guidelines were developed to help Extension educators navigate digital spaces and delivery, allowing them to implement DTHCS as closely as possible to the in-person events. As a result, DTHCS were delivered through online platforms such as Webex, Microsoft Teams, Zoom, and Facebook. This transition reduced outreach and participation. Additionally, participants may have experienced difficulties with food availability and access during this time, which could have impacted our findings.

Thus, the positive outcomes in nutrition knowledge and confidence in healthy cooking documented in our study, along with support from existing research, suggest that cooking interventions emphasizing healthy food preparation could play a significant role in promoting healthier lifestyles. For our study, while we focused on assessing nutrition knowledge and healthy cooking confidence, future research should explore the broader impact of cooking programs on various aspects of health and well-being. Existing evidence suggests that such programs can lead to improvements in diet quality, enhanced nutrient intake, positive changes in eating behaviors, and increased enjoyment in cooking [40,41,42].

## 5. Conclusions

Through our statewide implementation of DTHCS, our study documented the effectiveness of a community-based cooking program in promoting positive healthy food behaviors. Nutrition and culinary education can play a vital role in addressing the rising prevalence of obesity and associated diet-related chronic conditions. Enhancing cooking skills and nutrition knowledge can boost participants’ confidence in planning healthy meals, consuming more home cooked meals, and selecting healthy and affordable ingredients. Subsequently, this may lead to increased consumption of nutritionally superior foods, such as whole grains, fruits, vegetables, and lean protein at each meal, along with reduced reliance on fast foods and convenience foods, which may help lower health care costs associated with obesity and related complications [35,38,39].

The findings of this study can inform the planning and implementation of future community-based cooking interventions, particularly those with robust evaluation strategies. Further research is needed to assess the long-term impact of cooking schools on nutrition and food behaviors. Cooking programs like DTHCS, offered in group settings, have the potential to foster social connections, strengthen community support, and enhance both physical health and mental well-being [20,43,44]. Health care providers and dietitians can collaborate with state extension agencies to promote long-term health outcomes by encouraging at-risk individuals to enhance their nutrition knowledge and culinary skills through participation in cooking schools. Extension programs can be replicated in other states, broadening their reach and impact. Additionally, alternatives such as online cooking demonstrations and mobile cooking schools can expand accessibility and ensure greater participation.

## Figures and Tables

**Table 1:** Dinner Tonight Healthy Cooking School (DTHCS) data description and participant characteristics (n = 7932).

Participant Characteristics		n (%)
Age (years)	<18	318 (4.0)
	18–24	302 (3.8)
	25–34	950 (12.0)
	35–44	1225 (15.4)
	45–54	1491 (18.8)
	55–64	1485 (18.7)
	≥65	1892 (23.9)
	n/a	269 (3.4)
Gender	Male	1812 (22.8)
	Female	5804 (73.2)
	n/a	316 (4.0)
Race/Ethnicity	White	3488 (44.0)
	African American	650 (8.0)
	Hispanic	3204 (40.4)
	Other *	311 (3.9)
	n/a	279 (3.5)
Number of members in the household	Single-person household	1049 (13.2)
	Small household (2–3 members)	3828 (48.3)
	Medium household (4–5 members)	1808 (22.8)
	Large household (≥6 members)	402 (5.1)
	n/a	845 (10.7)
Texas Region	Central	527 (6.6)
	East	2526 (31.8)
	North	276 (3.5)
	South	3005 (37.9)
	Southeast	1468 (18.5)
	West	130 (1.6)

n/a = Not available due to missing data, * includes Asian, American Indian/Alaska Native, Native Hawaiian or Pacific Islander, multiracial, or other.

**Table 2:** Self-reported health, physical activity, and nutrition behaviors of Dinner Tonight Healthy Cooking School (DTHCS) participants (n = 7932).

Participant Self-Reported Health Behaviors		n (%)
Perception of general health	Excellent/very good/good	6104 (77.0)
	Fair/poor	1630 (20.5)
	n/a	198 (2.5)
Engage in moderate and/or vigorous physical activity in the past month	Yes	4779 (60.2)
	No	2625 (33.1)
	n/a	528 (6.7)
Meals consumed outside the home	Rarely/never (≤2 meals per week)	3375 (42.5)
	Occasionally (3–5 meals per week)	2505 (31.6)
	Frequently (6–9 meals per week)	576 (7.3)
	Very frequently (≥10 meals)	464 (5.8)
	n/a	1012 (12.8)
Amount spent each week on meals consumed outside the home	Low spending (<USD 25/week)	1729 (21.8)
	Moderate spending (USD 26–USD 75/week)	2687 (33.9)
	High spending (USD 76–USD 150/week)	1688 (21.3)
	Very high spending (>USD 150/week)	514 (6.5)
	n/a	1314 (16.6)
Portion of dinner plate filled with fruits and vegetables	More than 1/2	3140 (39.6)
	Less than 1/2	4512 (56.9)
	n/a	280 (3.5)
Fruit consumption	≥3 times a day	1027 (12.9)
	<3 times a day	6537 (82.4)
	n/a	368 (4.6)
Vegetable consumption	≥3 times a day	1498 (18.9)
	<3 times a day	6063 (76.4)
	n/a	371 (4.7)
Regular soda consumption	≤3 times a week	5808 (73.2)
	>3 times a week	1745 (22.0)
	n/a	379 (4.8)
Sweetened beverage consumption	≤3 times a week	5422 (68.4)
	>3 times a week	2005 (25.3)
	n/a	505 (6.4)

n/a = Not available due to missing data.

**Table 3:** Changes in participants’ self-reported nutrition knowledge and confidence level in healthy food preparation before and after attending Dinner Tonight Healthy Cooking School (DTHCS).

**Changes in Nutrition Knowledge** **(1 = poor; 2 = fair; 3 = good; 4 = excellent)**	**n**	**Before** **Mean (SD)**	**After** **Mean (SD)**	***p***
Benefits of meal planning	6800	2.73 (0.81)	3.50 (0.58)	<0.001
Sodium content of foods	6750	2.64 (0.84)	3.44 (0.64)	<0.001
Fat content of foods	6726	2.63 (0.81)	3.43 (0.63)	<0.001
Calorie content of foods	6736	2.58 (0.83)	3.41 (0.63)	<0.001
Impact of food on health	6739	2.82 (0.80)	3.57 (0.57)	<0.001
**Changes in Confidence Level** **(1 = none; 2 = low; 3 = moderate; 4 = high)**	**n**	**Before** **Mean (SD)**	**After** **Mean (SD)**	***p***
Meal planning for healthy eating	6732	2.77 (0.78)	3.58 (0.55)	<0.001
Modifying recipes to reduce calories	6702	2.64 (0.79)	3.53 (0.59)	<0.001
Modifying recipes to reduce sodium	6684	2.65 (0.81)	3.53 (0.60)	<0.001
Modifying recipes to change or reduce fat	6664	2.66 (0.81)	3.53 (0.60)	<0.001
Selecting fruits and vegetables	6611	2.83 (0.74)	3.62 (0.56)	<0.001
Safely storing meats	6550	2.86 (0.80)	3.58 (0.61)	<0.001
Safely preparing fruits, vegetables, and meats	6598	2.89 (0.78)	3.64 (0.56)	<0.001

## Data Availability

The datasets presented in this article are not readily available because they are managed by the Office of Data and Accountability at Texas A&M AgriLife Extension Service. The data presented in this study are available on request from the corresponding author.

## Abbreviations

The following abbreviations are used in this manuscript:

DTHCS	Dinner Tonight Healthy Cooking School
ODA	Office of Data and Accountability
